# Functional connectivity in cognitive control networks mitigates the impact of white matter lesions in the elderly

**DOI:** 10.1186/s13195-018-0434-3

**Published:** 2018-10-27

**Authors:** Gloria Benson, Andrea Hildebrandt, Catharina Lange, Claudia Schwarz, Theresa Köbe, Werner Sommer, Agnes Flöel, Miranka Wirth

**Affiliations:** 1 NeuroCure Clinical Research Center, Department of Neurology, Charité – Universitätsmedizin Berlin, Freie Universität Berlin, Humboldt-Universität zu Berlin, Berlin Institute of Health, Berlin, Germany; 2 Department of Psychology, University Medicine Greifswald, Greifswald, Germany; 3 Department of Psychology, Humboldt-Universität zu Berlin, Berlin, Germany; 4 Department of Nuclear Medicine, Charité – Universitätsmedizin Berlin, Freie Universität Berlin, Humboldt-Universität zu Berlin, Berlin Institute of Health, Berlin, Germany; 5 Department of Psychiatry, McGill University, Montreal, Quebec Canada; 6 Douglas Mental Health University Institute, Studies on Prevention of Alzheimer’s Disease Centre, Montreal, Quebec Canada; 7 Department of Neurology, University Medicine Greifswald, Greifswald, Germany; 8 Center for Stroke Research Berlin, Charité – Universitätsmedizin Berlin, Freie Universität Berlin, Humboldt-Universität zu Berlin, Berlin Institute of Health, Berlin, Germany

**Keywords:** White matter lesions, Functional connectivity, Reserve, Protective factors, Cognitive control networks

## Abstract

**Background:**

Cerebrovascular pathology, quantified by white matter lesions (WML), is known to affect cognition in aging, and is associated with an increased risk of dementia. The present study aimed to investigate whether higher functional connectivity in cognitive control networks mitigates the detrimental effect of WML on cognition.

**Methods:**

Nondemented older participants (≥ 50 years; *n* = 230) underwent cognitive evaluation, fluid-attenuated inversion recovery (FLAIR) magnetic resonance imaging (MRI), and resting state functional magnetic resonance imaging (fMRI). Total WML volumes were quantified algorithmically. Functional connectivity was assessed in preselected higher-order resting state networks, namely the fronto-parietal, the salience, and the default mode network, using global and local measures. Latent moderated structural equations modeling examined direct and interactive relationships between WML volumes, functional connectivity, and cognition.

**Results:**

Larger WML volumes were associated with worse cognition, having a greater impact on executive functions (β = −0.37, *p* < 0.01) than on memory (β = −0.22, *p* < 0.01). Higher global functional connectivity in the fronto-parietal network and higher local connectivity between the salience network and medial frontal cortex significantly mitigated the impact of WML on executive functions, (unstandardized coefficients: *b* = 2.39, *p* = 0.01; *b* = 3.92, *p* = 0.01) but not on memory (*b* = -5.01, *p* = 0.51, *b* = 2.01, *p* = 0.07, respectively). No such effects were detected for the default mode network.

**Conclusion:**

Higher functional connectivity in fronto-parietal and salience networks may protect against detrimental effects of WML on executive functions, the cognitive domain that was predominantly affected by cerebrovascular pathology. These results highlight the crucial role of cognitive control networks as a neural substrate of cognitive reserve in older individuals.

**Electronic supplementary material:**

The online version of this article (10.1186/s13195-018-0434-3) contains supplementary material, which is available to authorized users.

## Background

Cerebrovascular pathology, as quantified through white matter lesions (WML), is present in more than 50% of the elderly population [1]. WML are known to affect brain structure [2, 3] and cognitive performance [4–7], and have been associated with an increased risk of stroke and dementia [8]. Identifying beneficial lifestyle factors and brain mechanisms that protect against the negative effects of cerebrovascular pathology may be beneficial in preventing cognitive failure.

Cognitive dysfunction related to WML has been shown to be attenuated by protective lifestyle factors, such as educational attainment, cognitive enrichment, and physical activity [3, 9, 10], adding to the growing body of evidence for the concept of cognitive reserve (CR) [11]. Neuroimaging studies have extended the concept of CR to the level of functional brain mechanisms [12, 13]. It is suggested that those individuals with high CR have brain activation patterns that reflect higher neural efficiency, which may help maintain cognitive functions in the face of brain pathology [14]. While the reserve hypothesis has been well established in the context of WML with behavioral measures of CR [9, 15–17], the functional mechanisms within neural networks that may convey reserve in cerebrovascular pathology remain to be understood.

Some neuroimaging studies have provided an indication of active neuronal compensation in the context of WML. For example, in a working memory task, older individuals with higher WML volumes showed higher task-related brain activation across different levels of task complexity in anterior cingulate and middle frontal regions [18]. Fernández-Cabello et al. [19] found that older individuals with a high CR and a high WML load over-recruited fronto-parietal areas during task performance when compared with young individuals. These findings imply that higher neural capacity in brain regions subserving cognitive control could buffer the negative impacts of WML. More clarification is needed, however, on the moderating role of functional brain networks.

Recently, higher functional connectivity within major hubs of cognitive control networks have been proposed as neural correlates of CR [20]. Cognitive control networks are linked to reserve-associated protective factors [21], and have been suggested to play a compensatory role in the presence of early Alzheimer’s disease (AD) pathology [22]. More specifically, it was demonstrated that higher global connectivity in the fronto-parietal network [23] and higher local connectivity from the anterior cingulate cortex (a central hub of the salience network) [24, 25] may offer protection against the detrimental effects of age-related neuropathology. All together, these results motivated us to choose cognitive control networks, the fronto-parietal and the salience network, to examine reserve mechanisms and their moderating role in cerebrovascular pathology.

**Fig. 1:**
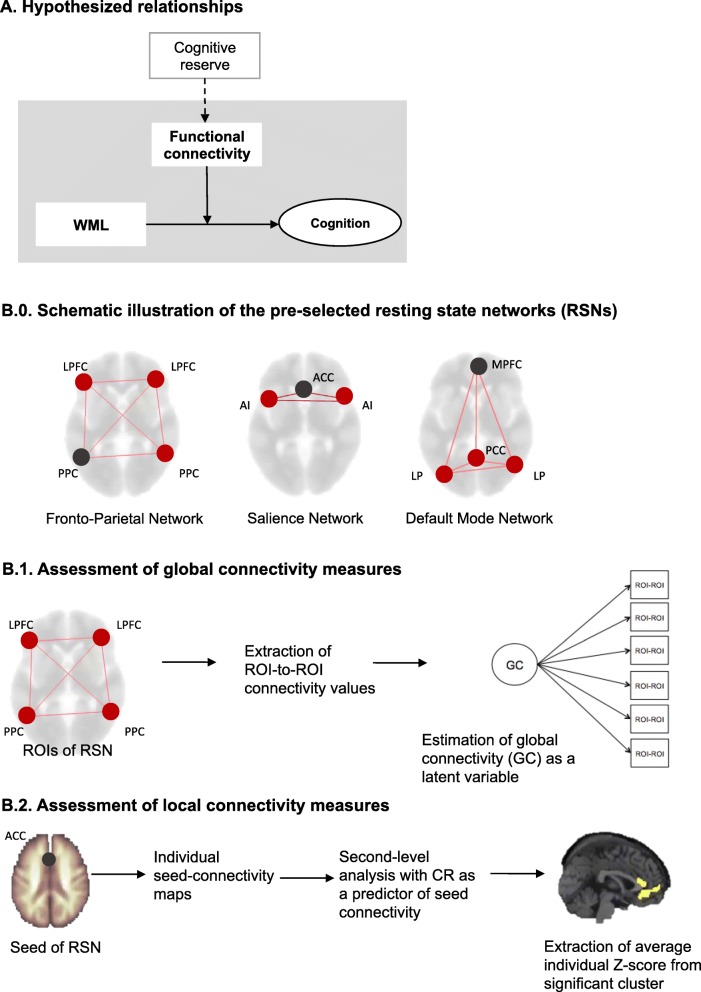
Panel A: Hypothesized relationships. The relationships analyzed in this study are shaded in gray. Functional connectivity, as a proxy of cognitive reserve (CR), may act as a moderator between white matter lesions and cognition. Panel B.0: Regions of interest (ROIs) for each resting state network as provided by CONN atlas. ROIs selected as seeds in the local connectivity measure are presented in grey. Panel B.1: Schematic representation of the assessment of global connectivity measures. Panel B.2: Assessment of local connectivity measure with our behavioral measure of CR indicated by years of education, premorbid intelligence, and lifestyle index. ACC anterior cingulate cortex, AI anterior insula, LP lateral parietal, LPFC lateral prefrontal cortex, MPFC medial prefrontal cortex, PCC posterior cingulate cortex, PPC posterior parietal cortex, WML white matter lesions

In the present study, we investigate whether resting state functional connectivity in cognitive control networks, as a proxy of CR, plays a role in mitigating the negative effect of cerebrovascular pathology on cognitive performance (Fig. 1, panel A). To this end, we assessed the relationships between the extent of WML (WML load), cognition, and functional connectivity using structural equation modeling (SEM) and tested for moderation effects in a sample of 230 nondemented individuals. We hypothesized the following: 1) a detrimental effect of WML on cognitive domains, such as executive functions and memory [6]; and 2) a moderating role of global and local functional connectivity in the fronto-parietal and salience networks, with the default mode network as control. More precisely, we expected that the negative relationship between WML load and cognitive performance would be reduced in individuals with higher levels of functional connectivity.

## Methods

### Participants

In total, 230 nondemented older participants, healthy older individuals (*n* = 140), and individuals with mild cognitive impairment (MCI; *n* = 90) were included in this study. Participants were aged between 50 and 80 years and were native German speakers. The healthy older individuals were recruited from the general community via advertisement. The Mini-Mental State Examination (MMSE) [26] was used to exclude pre-existing cognitive impairment (a score < 26 led to exclusion). Amnestic MCI patients were recruited from the memory clinic of the Department of Neurology at the Charité University Hospital, Berlin, and a Neurology specialist practice in Berlin (Dr. J. Bohlken). Individuals with MCI were diagnosed according to the standardized Mayo Clinic criteria [27]. Exclusion criteria for both groups included severe medical, neurological, or psychiatric disease. Detailed information of the samples has been provided previously [28, 29].

### Neuropsychological testing

Participants underwent a full neuropsychological test battery focused on a variety of cognitive domains. Based on their relevance for the present research questions, the following psychometric tests were selected for further analysis: learning and memory performance was evaluated by the German version of the Auditory Verbal Learning Test (VLMT) [30], providing subscores for learning ability (total immediate recall), delayed recall, and recognition. Executive functions were measured by the Trail Making Test (TMT) version A and B [31, 32] and the interference score from the Stroop Color-Word interference test [33]. In addition, working memory and language abilities, respectively, were measured using the forward and backward digit span conditions from the Wechsler Digit span task [34] and phonemic and alternating word fluency [35].

### Acquisition preprocessing and analysis of the neuroimaging data

#### Magnetic resonance imaging (MRI) acquisition

Scans were acquired using a 3-Tesla Magnetom Trio (Tim Trio; Siemens AG, Erlangen, Germany) at two different sites using identical imaging protocols. T1-weighted images were acquired with magnetization-prepared rapid acquisition gradient-echo (MPRAGE) with the following parameters: repetition time (TR = 1900 ms; TE = 2.52 ms; 192 sagittal slices; size = 1.0 × 1.0 × 1.0 mm^3^; flip angle = 9°). Functional scans were obtained at rest using T2*-weighted EPI sequence (TR = 2300 ms; TE = 30 ms; 34 slices; size = 3.0 × 3.0 × 4.0 mm^3^; flip angle = 90°). Subjects were instructed to keep their eyes closed and not think of anything in particular. Fluid attenuated inverse recovery (FLAIR) T2-weighted images (TR = 8000 ms; TE = 100 ms; 2370 inversion time; 232 × 256 matrix size = 0.86 × 0.86 × 5.0 mm^3^; flip angle = 130°; slice gap = 5.0 mm) were acquired to measure WML. Neuroimaging measurements and neuropsychological test sessions were obtained in close proximity (mean time delay, 12.9 days; range, 1–40 days).

#### Assessment of WML and vascular risk

Total WML volumes were segmented automatically using the FLAIR images and the “lesion growth algorithm” of the lesion segmentation toolbox (LST) under the freely available Statistical Parametric Mapping (SPM) software package (version SPM8, Wellcome Trust Centre for Neuroimaging, Institute of Neurology, UCL, London, UK; [36]). Processing and parameter settings (kappa = 0.30, binarization threshold = 0.50) were exactly as described previously [37]. The total WML volume was obtained by multiplying the number of WML voxels according to the binary WML map by the voxel volume. For each subject, WML volume ratio was computed as the volume of WML divided by the total intracranial (TIV) volume. Individual TIV was assessed with the Tissue Volumes utility in SPM 12 (Wellcome Trust Centre for Neuroimaging, London, UK; www.fil.ion.ucl.ac.uk/spm). It computes the total by summing the volumes of grey matter, white matter, and cerebrospinal fluid (CSF) from the corresponding segmented images [38]. Frequency maps were calculated for each group, both separately and for the entire sample. To this aim, the frequency (i.e., number of participants with WML in specific voxels relative to total number of participants) was computed voxel-wise based on binarized WML segmentation maps previously warped to the anatomic Montreal Neurologic Institute reference space.

In addition, we computed the validated Framingham risk index of cardiovascular disease (CVD) as a combined measure of vascular risk to validate the WML measure based on the present sample [39]. This measure involves age, sex, total cholesterol, high-density lipoprotein (HDL) cholesterol, systolic blood pressure, medical history of diabetes, treatment for hypertension, and smoking status.

#### Preprocessing and analysis of resting state functional MRI

The publicly available CONN Functional Connectivity Toolbox version 17C (www.nitrc.org/projects/conn), in conjunction with SPM 12 (Wellcome Department of Cognitive Neurology, London, UK; www.fil.ion.ucl.ac.uk/spm), was used to perform all preprocessing steps [40]. In detail, we used the default preprocessing pipeline: raw functional images were slice-time corrected, realigned (motion corrected), and coregistered to each participant’s MPRAGE image. Images were then normalized to the Montreal Neurological Institute (MNI) standard space and spatially smoothed with an 8-mm Gaussian filter. Identification of outlier scans was performed using Artifact Detection Tools (http://www.nitrc.org/projects/artifact_detect; [40]). Specifically, this regresses out scans as nuisance covariates in the first-level analysis exceeding 3 standard deviations (SD) in mean global intensity and frame-to-frame differences exceeding 0.5 mm (combination of translational and rotational displacements). There were no significant differences between the two groups in the number of outlier scans (*p* = 0.6) or mean motion (*p* = 0.2); details in Additional file 1 (Table S2). Resting state images were band-pass filtered (0.008–0.09 Hz) and corrected with the implemented component correction (CompCor) strategy [41], including the removal of white/CSF time series, motion, and artifact-outlier regressors, to reduce the influence of blood oxygen level-dependent (BOLD) signals unrelated to neural activity. This approach limits the influence of confounds such as head motion, peripheral physiology, and other imaging artifacts.

#### Functional connectivity assessment

Functional connectivity was assessed within preselected cognitive control networks, namely the fronto-parietal network and the salience network, using global and local connectivity measures (Fig. 1, panel B.0). The default mode network was added for comparison reasons. Global network connectivity was estimated within each resting state network, using the atlas network region(s) of interest (ROI) (8-mm radius spheres) provided by CONN. ROI-to-ROI connectivity values (Fisher-transformed correlation coefficients) at false discovery rate (FDR)-corrected level were extracted for each ROI pair within each network [40] The ROI-to-ROI connectivity values were used as indicators of latent variables (one for each network) in SEM (see below) for estimating global functional connectivity within each resting state network (Fig. 1, panel B.1).

Local network connectivity was assessed within each resting state network by extracting those brain regions that significantly correlated with our behavioral measure of CR (explained in detail below), similar to previous approaches [24]. Individual connectivity maps were derived using seed-to-voxel analyses from CONN (Fig. 1, panel B.2). Whole brain correlational maps were generated by extracting the mean resting state BOLD time course for each seed ROI and calculating the Fisher-transformed correlation coefficients with the BOLD time course throughout the whole brain. For each network the following ROIs (Fig. 1, Panel B.0) were used as seeds: fronto-parietal network (left posterior parietal cortex (LPPC): –46,–58,49), salience network (anterior cingulate cortex (ACC): 0,22,35), and default mode network (medial prefrontal cortex (MPFC): 1,55,−3). We chose these seeds as they are characterized as core network hubs [42, 43] and are areas involved in reserve-related functional connectivity findings [20, 24, 44]. Individual connectivity maps were then subjected to voxel-wise second-level analysis with our behavioral measure of CR as a predictor of local connectivity related to reserve. Significant clusters were extracted at a cluster-level threshold of *p* < 0.05, FDR-corrected for multiple comparison, and a voxel-level threshold of *p* < 0.005. Finally, the average *Z* scores across each individual cluster for each subject were used as a local connectivity measure.

### Modeling procedure and measurement models

The SEM builds upon multiple observed variables to estimate latent variables. We used the software Mplus for the purpose of modeling [45]. Structural equational modeling allows estimation of the relationship between observed variables and the latent variable they intend to measure (measurement models), and relationships between multiple latent variables (structural models). The advantage of latent variables is that they represent the shared variance among multiple observed variables that are conceivable realizations of cognitive ability as a construct. Thus, latent variables are adjusted for measurement error and for the specificity of applied assessment methods in a given study. Due to this adjustment, results based on latent variables are generalized above measurement methods.

To that end, we established the best fitting measurement models, separately for cognition, CR, and each resting state network, aiming to estimate the number and structure of latent variables that are necessary to explain the relationships across all these measured variables at the levels of brain and behavior.

### Cognition, connectivity, and cognitive reserve estimate models

The cognitive model included a latent variable of global cognition (*G*), indicated by all selected psychometric tests. Above *G*, executive functions and memory were modeled as nested latent variables under *G*. As mentioned previously, executive functions were indicated by TMT versions A and B, and Stroop interference, while memory was indicated by VLMT total immediate recall, delayed recall, and recognition. The first model postulated *G* with the specific nested variables added in a stepwise fashion and testing for model fit improvement through latent variable addition. For subsequent analyses of specific relationships within a given cognitive domain, the latent variables memory and executive functions were assessed as separate latent factors. Additional file 1 (Table S1) provides the fit of all estimated measurement models.

For each resting state network, global network connectivity was estimated as a latent variable, as indicated by the functional ROI-to-ROI connectivity among the major network nodes. To account for the shared variance of pairs of ROI-to-ROI connectivity values, some residual covariance between connectivity indicators was introduced (i.e., MPFC-right lateral parietal (LP) with MPFC-left LP). The model fit for each resting state network is provided in Additional file 1 (Table S1).

Finally, we estimated a behavioral measure of CR as a latent variable based on the following observed measures: years of education, premorbid intelligence, and a combined measure of self-reported healthy lifestyle behaviors (referred to as lifestyle index). Premorbid verbal intelligence was assessed by the German multiple vocabulary test [46]. The lifestyle index included a sum score of body mass index, dietary habits, physical exercise, smoking, and alcohol consumption, described in detail elsewhere [47, 48]. A high lifestyle index score indicated normal weight, never smoking, intense physical activity, moderate alcohol consumption, and a dietary pattern rich in fruits, vegetables, and whole-grain products, as well as unsaturated fatty acids.

Several statistical test and fit indices were used for assessing model fit: the ratio between χ^2^ and degrees of freedom (χ^2^/df ratio < 2), root-mean square error of approximation (RMSEA) ≤ 0.08, standard root mean square residual (SRMR) ≤ 0.05, and comparative fit index (CFI) ≥ 0.95 [49]. Competing models were compared by evaluating the difference of their likelihoods, using the χ^2^-difference test. Missing data were dealt with by the full information maximum likelihood (FIML) algorithm, as implemented in Mplus (details of missing data provided in Table 1).

**Table 1:** Characteristics of the study group showing means, standard deviation, and range of the total sample and dichotomized by group

	Total sample	HO	MCI	
*N* (*n* women)	230 (115)	140 (71)	90 (44)	
Age (years)	65.2 ± 7.6 (50–80)	63 ± 6.9 (50–79)	68.6 ± 7.5 (50–80)	**
APOE4 carrier (%)	71 (30%) (*n* = 228)	27 (19%)	44 (49%)	**
WML/TIV	0.17 ± 0.37 (0–2.8) (*n* = 229)	0.11 ± 0.25 (0–1.5)	0.28 ± 0.48 (0–2.8)	**
Cognitive reserve				
Education	15.8 ± 3.3 (6–29)	16 ± 3.1 (10–25)	15 ± 3.7 (6–29)	
MWT	31.9 ± 2.7 (21–37)	32.4 ± 2 (24–37)	31.1 ± 3 (21–37)	
Lifestyle index	16.2 ± 2.6 (7–22) (*n* = 139)	16.3 ± 2.5 (7–20)	16.1 ± 2.7 (9–22)	
Cognition				
MMSE	28.7 ± 1.2 (24–30)	29.0 ± 1.1 (26–30)	28.3 ± 1.4 (24–30)	**
G factor score	–	0.35 ± 0.8 (−1.6 to 1.8)	−0.55 ± 0.9 (−1.3 to 2.6)	**
Executive function factor score	–	0.25 ± 0.6 (−1.2 to 2.7)	−0.39 ± 1.1 (−1.1 to 4.7)	**
Memory factor score	–	0.33 ± 0.8 (−1.9 to 1.7)	−0.52 ± 0.9 (−2.5 to 1.3)	**

Numbers are expressed as mean ± standard deviation, the ranges shown in parenthesis

Cognition variables are the factor scores estimated in the latent variable models. Because the latent variables of cognition were scaled by standardization (*M* = 0; σ = 0), they are not displayed for the whole group. Group-specific average factor score can be thus interpreted as deviations from the whole sample average

White matter lesions (WML) data were missing in one participant (mild cognitive impairment (MCI): *n* = 1), and lifestyle index was missing in 91 participants (healthy older (HO): *n* = 61, MCI: *n* = 12)

*APOE4* apolipoprotein E, *MMSE* Mini-Mental State Examination, *MWT* German multiple vocabulary test, *TIV* transcranial intracranial volume

**p < 0.01, independent sample test for continuous variables and chi-square test for categorical variables

Additional statistical analyses were conducted with SPSS (version 24) to evaluate the reproducibility of our results when simplified modeling is applied. Restricted regression models were computed to control for covariates such as age and total grey matter volume. Cook distance (> 1) was used to detect potential influential cases [50].

#### Statistical analysis

The analysis objectives of this study can be summarized as follows. First, the direct effect of WML on cognitive performance (*G*, memory, and executive function in the overall cognitive model) was estimated. Next, we tested whether functional connectivity (global and local measures) within each resting state network moderated the relationship between WML and executive function and memory, respectively (Fig. 1, panel A). To this aim, we estimated latent moderated structures implemented in Mplus [51]. The moderation was assessed through an interactive term, modeled by the product of WML and functional connectivity values, respectively. Latent variables of executive functions and memory were then regressed onto WML volumes, functional connectivity measures, and their interactive term.

All models were estimated based on the whole sample of nondemented individuals, which includes healthy older individuals and individuals with MCI. This was done to include a larger spectrum of individuals in whom there is sufficient pathology to cause cognitive impairment. We furthermore conducted sensitivity analysis using multigroup structural equation modeling to explore, post hoc, the significant moderation effects within each group (healthy older individuals vs. MCI). Thus, latent interactions for testing moderation effects of functional connectivity on the relationship between WML and cognition were estimated separately, but simultaneously for healthy older individuals vs MCI. Because the model included a latent interaction between functional connectivity and the relationship between WML and cognition, such a model can be established as a latent interaction model using the mixture modeling framework of Mplus. In this framework the groups (healthy older individuals and MCI) are treated as known latent classes whereas the latent interaction is estimated simultaneously, but separately for the two classes (participant groups).

Factor scores, extracted from each latent variable, were used to visualize selected interactive relationships from regression models to better understand their directionality using the R package Jtool (available at: https://cran.r-project.org/web/packages/jtools/). Centered mean predicted scores were estimated for executive function and memory on two levels of low and high (–1 SD and +1 SD) functional connectivity measures. Finally, a mediation model was included to further validate our WML measure with CVD risk score and cognition [51].

## Results

### Sample characteristics

**Fig. 2:**
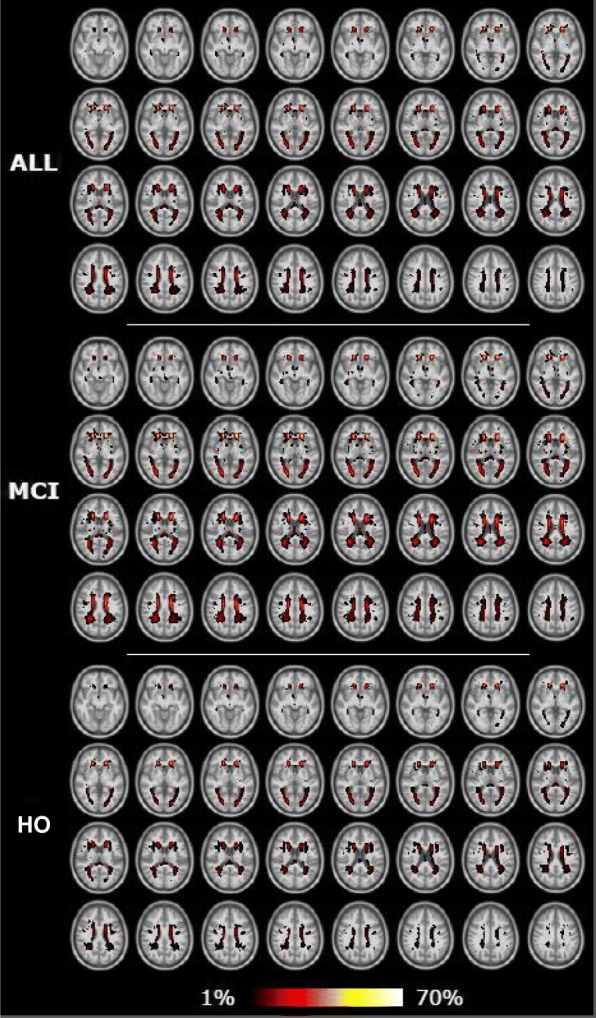
White matter lesion frequency maps for the entire sample and dichotomized by group in anatomic Montreal Neurologic Institute reference space. HO healthy older, MCI mild cognitive impairment

Descriptive information on the total sample of nondemented older participants as well as participants dichotomized by group is provided in Table 1. The MCI group had a higher frequency of APOE4 carriers, was significantly older, and performed significantly worse on the cognitive measures (cognitive scores for each test are provided in Additional file 1: Table S3). The groups did not otherwise differ demographically. The lesion frequency maps of participants for the total sample and for each group category are provided in Fig. 2. The figure shows lesions situated predominately in periventricular areas with more pronounced lesions in the frontal regions.

### Relationships between WML and cognition

**Fig. 3:**
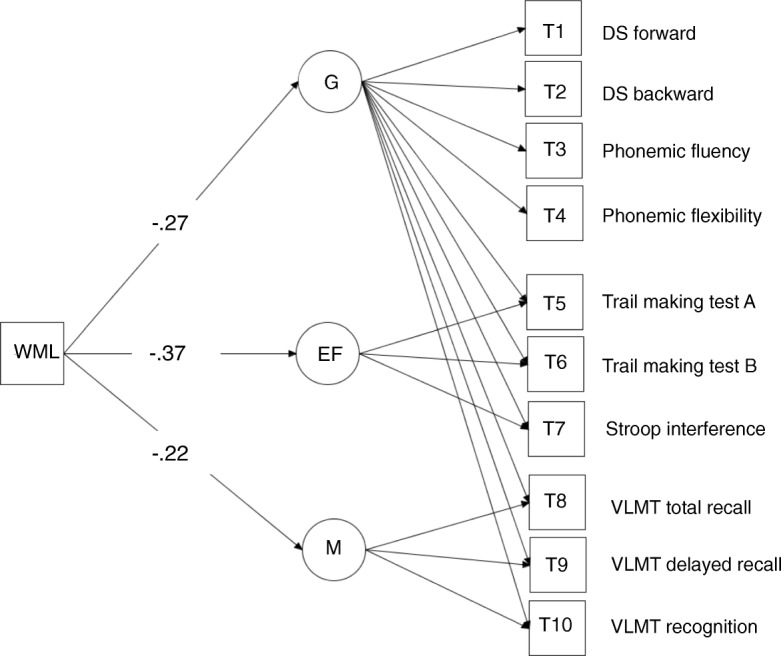
Schematic representation of the structural equation model with path coefficients showing the direct effect of white matter lesions (WML) on the latent variables of global cognition (G), executive functions (EF), and memory (M). Note that the executive function tests were inverted to indicate better performance with higher scores. DS digit span, VLMT Auditory Verbal Learning Test

Structural equational modeling confirmed negative relationships between WML volumes and cognitive performance (Model fit: χ^2^ = 73.06, df = 36, χ^2^/df = 2.02, RMSEA = 0.06, SRMR = 0.04, CFI = 0.96). Larger WML volumes were significantly related to lower *G* (β_1_ = −0.27, *p* < 0.01), having an even higher impact on executive functions (β_2_ = −0.37, *p* < 0.01) compared with memory (β_3_ = −0.22, *p* < 0.01) (Fig. 3). These effects remained significant when controlling for age and grey matter volume.

In a follow-up analysis, we added CVD risk to the model defined as a predictor of WML volumes and cognition to further validate our WML measure (model fit: χ^2^ = 74, df = 43, χ^2/^/df = 1.72 RMSEA = 0.05, SRMR = 0.04, CFI = 0.97). CVD risk was related to worse cognition (executive functions β = −0.30, *p* < 0.01 and memory β = −0.26 *p* < 0.01). This relationship was mediated by WML load, as indicated by a significant indirect effect (*β* = −0.12, confidence interval (CI) −0.244 to −0.001, and *β* = −0.08, CI −0.154 to −0.002) for executive functions and memory, respectively. Finally, there was no significant relationship between CVD risk factor and our behavioral measure of CR (*r* = −0.046, *p* = 0.49).

### Relationships between WML, connectivity, and cognition

#### Global connectivity

**Fig. 4:**
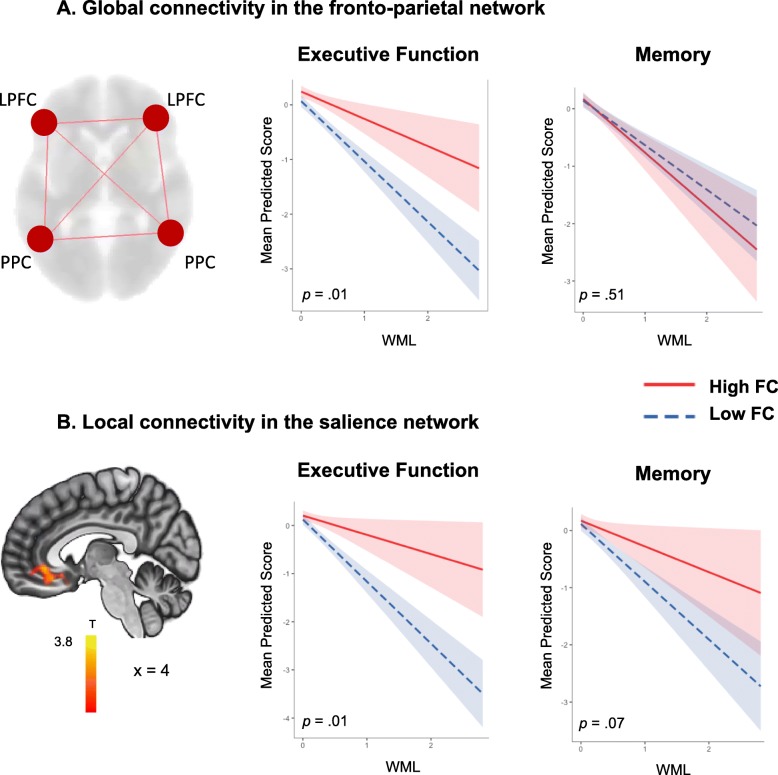
Moderations of functional connectivity on the effect of white matter lesions (WML) on cognition. Regression line plots showing the mean predicted scores of cognition on two levels of functional connectivity (FC), high (1 SD) and low (–1 SD) on WML. **a** The negative impact of WML on executive functions was reduced in individuals with higher levels of global functional connectivity in the fronto-parietal network. A similar moderation effect was not found for memory. **b** The local connectivity cluster from the salience network extracted as the multiple regression between our behavioral measure of cognitive reserve and the anterior cingulate cortex seed (*p* < 0.005, FDR corrected). Regression line plots show a significant moderation of local functional connectivity in the salience network on the negative impact of WML on executive functions and a trend for memory. Shaded area indicates 80% confidence intervals; *p* values of the interaction terms are displayed for each graph. LPFC lateral prefrontal cortex, PPC posterior parietal cortex

First, we modeled global functional connectivity as a latent variable for each resting state network. The model fit for each resting state network is provided in Additional file 1 (Table S1). All standardized factor loadings were statistically significant. Next, we tested whether global connectivity measures moderated the relationship between WML and cognition. Global connectivity of the fronto-parietal network showed a significant moderating effect on the relationship between WML and executive function (nonstandardized coefficient: *b* = 2.39, *p* = 0.01), but not for memory (nonstandardized coefficient: *b* = −5.01, *p* = 0.51). Specifically, the negative impact of WML on executive functions was reduced in individuals with higher levels of global connectivity in the fronto-parietal network (Fig. 4a). No significant interactions between WML and global connectivity were found for the salience network (nonstandardized coefficients: *b* = 0.24, *p* = 0.89; *b* = 0.15, *p* = 0.64) and the default mode network (nonstandardized coefficients: *b* = 0.19, *p* = 0.55; *b* = 0.05, *p* = 0.80) for executive functions and memory, respectively.

#### Local connectivity

For our local connectivity measure, we first used the behavioral measure of CR (modeled as a latent variable) as a predictor of local connectivity. Specific regions within the fronto-parietal network, the salience network, and the default mode network were positively related with CR at the given statistical threshold (with clusters in Additional file 1: Table S4). When testing for moderation effects, local connectivity in the salience network (cluster shown in Fig. 4b, medial frontal cortex, cingulate gyrus; peak voxel MNI: −12 + 38–4, *p* < 0.01) showed a significant moderation effect on the relationship between WML volumes and executive functions (nonstandardized coefficient: *b* = 3.92, *p* = 0.01) and a trend for memory (nonstandardized coefficient: *b* = 2.01, *p* = 0.07). The negative impact of WML on executive functions was reduced in individuals with higher local connectivity in the ACC (Fig. 4b). No significant interactions between WML and local connectivity in the fronto-parietal network (nonstandardized coefficient: *b* = −0.41, *p* = 0.85; *b* = −1.10, *p* = 0.31) and the default mode network (non-standardized coefficient: *b* = −0.82, *p* = 0.52; *b* = 0.38, *p* = 0.70) were found for executive functions and memory, respectively (data not shown). All the effects reported above remained significant after controlling for age and grey matter volume.

#### Post-hoc multigroup analysis

Multigroup SEM examined the associations (moderations) across each diagnostic group, where the groups are handled as a higher-order moderator variable and interaction effects of functional connectivity on the relationship between WML and cognition are estimated within groups (see the explanation in the methods section). In the MCI sample, the moderating effect for global connectivity of the fronto-parietal network between WML and cognition remained significant for executive function (nonstandardized coefficient: *b* = 3.10, *p* < 0.01). Likewise, the moderating effect of local connectivity within the salience network remained significant for both executive function and memory (nonstandardized coefficient: *b* = 8.97, *p* < 0.01; *b* = 5.65, *p* < 0.01, respectively). However, these moderating effects were not statistically substantial in the sample of healthy older individuals, for either the global fronto-parietal connectivity on executive function (nonstandardized coefficient: *b* = 0.96, *p* = 0.31), or for the local connectivity of the salience network (nonstandardized coefficient: *b* = 1.28, *p* = 0.35; *b* = −0.34, *p* = 0.78) executive functions and memory, respectively.

## Discussion

The present study evaluated the moderating impact of functional connectivity on the relationship between WML and cognitive performance in nondemented older individuals. Our results indicated that higher levels of functional connectivity in the fronto-parietal network and salience network in part mitigate the negative effect of WML on executive functions, the cognitive domain most affected by cerebrovascular pathology. Analyses were performed with SEM, allowing us to abstract from measurement error and task specificity [45]. Our results support the notion that higher functional connectivity in cognitive control networks may serve as protective neural mechanism that allow better preservation of cognitive ability in the presence of cerebrovascular pathology.

Our results are consistent with the established literature, suggesting an association between higher WML load and lower cognitive performance in the domains of both memory and executive functions [1, 7]. WML tend to primarily affect processing speed and executive tasks in older participants with Alzheimer’s disease, MCI, and normal cognition [5, 6, 52, 53]. Our results confirmed the stronger association with executive cognitive dysfunctions, with similar path coefficients as reported previously [17]. Although not always present [6], we found an association between lower memory performance and higher WML load, consistent with previous findings [5, 52]. The topography of WML (Fig. 2) show a higher frequency of lesions in frontal and periventricular regions, which is consistent with studies that report an association between WML frequency in these regions and decreased executive function and processing speed [6, 52]. In general, WML have been associated with a decline in cognitive domains linked to prefrontal cortex function and, to a lesser extent, with medial temporal lobe-associated memory tasks [4].

Consistent with our hypothesis, we found a significant moderating effect of the global functional connectivity in the fronto-parietal network. Thus, the negative impact of WML on executive functions was attenuated in individuals with higher global functional connectivity in this network. Our results are in line with previous findings that support the protective role of fronto-parietal network connectivity as a neural substrate of CR in both normal and pathological aging [54]. Higher functional connectivity (particularly in the left hub) has been associated with higher education and higher cognitive function in cognitively normal individuals and MCI patients [54] and has been shown to diminish the effect of Alzheimer’s disease pathology on cognition [23, 55]. Our results further converge with the previous findings of Franzmeier and colleagues [23, 44]. These authors have repeatedly found evidence for a compensatory effect of the global connectivity in the fronto-parietal network in Alzheimer’s disease pathology. Our results extend the evidence by demonstrating a protective role of the global fronto-parietal network against the detrimental impact of cerebrovascular pathology in the elderly.

At the local level, functional connectivity from the salience network showed a significant moderation on the impact of WML on cognition. More specifically, functional connectivity between the ACC (as seed) and the medial frontal cortex significantly mitigated the negative impact of WML on executive functions and, as a trend, this moderation effect was present for the memory domain. The regions involved in the local connectivity measure of the salience network (connectivity cluster in Fig. 4b) are in line with previous reports that show a positive correlation between connectivity from the ACC and the medial frontal cortex with higher levels of education and preserved cognitive performance in healthy elders [24]. Furthermore, a previous study [20] comparing MCI patients with low and high CR showed that the ACC was involved in regions showing connectivity changes at the local level. Our findings extend the possible beneficial effects of functional connectivity against WML to include the salience network regions.

Results from the post-hoc multigroup analysis showed the estimated interactions to be significant in the whole sample and in the MCI sample alone. There may not have been enough pathology in the healthy older group, compared with the MCI, to yield a moderating relationship of functional connectivity on cognition. The smaller sample size of the subgroups may have also led to insufficient power to identify the effect with the healthy control group only. Our findings nevertheless support the idea that compensatory mechanisms are pronounced at the prodromal disease stage, where more neuropathology is present [56].

Both the salience and the fronto-parietal network are considered as important cognitive control networks crucial for regulation and healthy brain functioning. The fronto-parietal network is important for flexibly regulating activity to other functional networks [42], just as the salience network is crucial for integrating input from various sources [57]. Both networks support successful cognition with increased functional hub connectivity linked to better cognition [25, 58]. Higher or more efficient functional connectivity in these networks may facilitate adaptive functional connectivity to other brain regions when neurodegenerative insults occur. Our results show that, indeed, functional neural mechanisms convey reserve in the presence of cerebrovascular pathology and substantiate the notion that cognitive control networks may play an important role in resilience mechanisms.

The detection of resilient or protective mechanism are of increased recent interest given the rapidly aging population [59, 60]. Functional mechanisms underlying reserve may be suitable targets for therapeutic intervention to prevent further cognitive decline. For example, combining cognitive training and noninvasive brain stimulation over task-relevant brain areas may offer a means for cognitive enhancement in older adults, as demonstrated both in healthy older adults [61] as well as in patients with MCI [62] (see also [63] for a recent review). The present study suggests that targeting hubs specifically involved in resilient mechanisms may provide an additional approach to protect cognitive function against age-related conditions in the elderly.

There are several caveats that must be taken into consideration when interpreting our results. First, although our measure of WML is reliable, our sample was prescreened for cerebrovascular disease and included individuals with MCI. Thus, the compensatory mechanisms of functional connectivity should be replicated in a sample with higher WML load. Second, our measure of global functional connectivity as a latent variable may be specific to our SEM analysis. Our findings need to be completed by other functional connectivity measures, such as inter-network functional connectivity and degree of centrality and extended to other intrinsic brain networks [64]. A future line of work might specifically explore inter-network functional connectivity in order to elucidate the relationship of functional connectivity between networks. Third, the present study focused on functional connectivity; however, structural measures of white matter tracts through diffusion tensor imaging (DTI) should also be tested for attenuation effects underlying reserve. Recent work has explored the disruption of tract-specific WML on the default mode network [65]. However, the fronto-parietal and salience networks and their moderation effects should also be explored in this modality. Fourth, WML represent only one entity of the umbrella term of cerebrovascular disease; other pathologies (i.e. lacunes, small infarcts and microbleeds) should also be considered. More pronounced effects could be observed by the incorporation of these pathologies into the model. Finally, longitudinal studies are necessary to assess the neuroprotective trajectories of functional connectivity and whether there are nonlinear relationships with the increase in further pathology.

## Conclusion

The results from the current study highlight the role of functional connectivity in cognitive control networks in attenuating the detrimental effects of cerebrovascular pathology in the elderly. Our findings shed light on neural mechanisms underlying reserve in the face of cerebrovascular pathology and suggest that the fronto-parietal network and the salience network may be suitable targets for early intervention strategies that aim to enhance CR in the elderly.

## Additional file

Additional file 1: **Table S1.** Measurement models. Note: given that the salience network and reserve model only contain three nodes/indicators, model fit cannot be provided as the model is just identified. However, factor loadings are considerably high between 0.53 and 0.81. CFI comparative fit index, RMSEA root mean square error of approximation, SRMR standard root mean square residual. **Table S2.** Movement parameters depicted as mean and standard deviation. Independent sample test for group differences. **Table S3.** Cognitive scores are depicted as mean and standard deviation (SD) with range for the entire sample and by group. **Table S4.** Results of seed-to-voxel based regression analysis of CR in each network seed. Coordinates are provided in MNI coordinates (*xyz*). Significant clusters were extracted at a cluster-level threshold of *p* < 0.05, FDR-corrected for multiple comparison, and a voxel-level threshold of *p* < 0.005. LP lateral parietal, ACC anterior cingulate cortex, MPFC medial prefrontal cortex. (DOCX 25 kb)

## Abbreviations

ACC: Anterior cingulate cortex
CR: Cognitive reserve
CVD: Cardiovascular disease
LP: Lateral parietal
MPFC: Medial prefrontal cortex
WML: White matter lesions

## References

[1] Prins ND, Scheltens P (2015). White matter hyperintensities, cognitive impairment and dementia: an update. Nat Rev Neurol.

[2] Raz Naftali, Rodrigue Karen M. (2006). Differential aging of the brain: Patterns, cognitive correlates and modifiers. Neuroscience & Biobehavioral Reviews.

[3] Wirth M, Haase CM, Villeneuve S, Vogel J, Jagust WJ (2014). Neuroprotective pathways: lifestyle activity, brain pathology, and cognition in cognitively normal older adults. Neurobiol Aging.

[4] Au R, Massaro JM, Wolf PA, Young ME, Beiser A, Seshadri S (2006). Association of white matter hyperintensity volume with decreased cognitive functioning: The Framingham Heart Study. Arch Neurol.

[5] Tullberg M., Fletcher E., DeCarli C., Mungas D., Reed B. R., Harvey D. J., Weiner M. W., Chui H. C., Jagust W. J. (2004). White matter lesions impair frontal lobe function regardless of their location. Neurology.

[6] Birdsill AC, Koscik RL, Jonaitis EM, Johnson SC, Okonkwo OC, Hermann BP (2014). Regional white matter hyperintensities: aging, Alzheimer’s disease risk, and cognitive function. Neurobiol Aging.

[7] Wirth M, Villeneuve S, Haase CM, Madison CM, Oh H, Landau SM (2013). Associations between Alzheimer disease biomarkers, neurodegeneration, and cognition in cognitively normal older people. JAMA Neurol.

[8] Debette S, Markus HS (2010). The clinical importance of white matter hyperintensities on brain magnetic resonance imaging: systematic review and meta-analysis. BMJ.

[9] Nebes R, Meltzer C, Whyte E, Scanlon J, Halligan E, Saxton J (2006). The relation of white matter hyperintensities to cognitive performance in the normal old: education matters. Neuropsychol Dev Cogn B Aging Neuropsychol Cogn.

[10] Saczynski JS, Jonsdottir MK, Sigurdsson S, Eiriksdottir G, Jonsson PV, Garcia ME (2008). White matter lesions and cognitive performance: the role of cognitively complex leisure activity. J Gerontol A Biol Sci Med Sci.

[11] Stern Y (2002). What is cognitive reserve? Theory and research application of the reserve concept. J Int Neuropsychol Soc.

[12] Bartrés-Faz D, Arenaza-Urquijo EM (2011). Structural and functional imaging correlates of cognitive and brain reserve hypotheses in healthy and pathological aging. Brain Topogr.

[13] Barulli D, Stern Y (2013). Efficiency, capacity, compensation, maintenance, plasticity: emerging concepts in cognitive reserve. Trends Cogn Sci.

[14] Stern Yaakov (2009). Cognitive reserve☆. Neuropsychologia.

[15] Dufouil C, Alpérovitch A, Tzourio C (2003). Influence of education on the relationship between white matter lesions and cognition. Neurol Int.

[16] Zieren N, Duering M, Peters N, Reyes S, Jouvent E, Hervé D (2013). Education modifies the relation of vascular pathology to cognitive function: cognitive reserve in cerebral autosomal dominant arteriopathy with subcortical infarcts and leukoencephalopathy. Neurobiol Aging.

[17] Brickman AM, Siedlecki KL, Muraskin J, Manly JJ, Luchsinger JA, Yeung LK (2011). White matter hyperintensities and cognition: testing the reserve hypothesis. Neurobiol Aging.

[18] Griebe Martin, Amann Michael, Hirsch Jochen G., Achtnichts Lutz, Hennerici Michael G., Gass Achim, Szabo Kristina (2014). Reduced Functional Reserve in Patients with Age-Related White Matter Changes: A Preliminary fMRI Study of Working Memory. PLoS ONE.

[19] Fernández-Cabello S, Valls-Pedret C, Schurz M, Vidal-Piñeiro D, Sala-Llonch R, Bargallo N (2016). White matter hyperintensities and cognitive reserve during a working memory task: a functional magnetic resonance imaging study in cognitively normal older adults. Neurobiol Aging.

[20] Serra L, Mancini M, Cercignani M, Di Domenico C, Spanò B, Giulietti G (2016). Network-based substrate of cognitive reserve in Alzheimer’s disease. J Alzheimers Dis.

[21] Cole M. W., Yarkoni T., Repovs G., Anticevic A., Braver T. S. (2012). Global Connectivity of Prefrontal Cortex Predicts Cognitive Control and Intelligence. Journal of Neuroscience.

[22] Elman JA, Oh H, Madison CM, Baker SL, Vogel JW, Marks SM (2014). Neural compensation in older people with brain amyloid-β deposition. Nat Neurosci.

[23] Franzmeier N, Duering M, Weiner M, Dichgans M, Ewers M (2017). Left frontal cortex connectivity underlies cognitive reserve in prodromal Alzheimer disease. Neurology.

[24] Arenaza-Urquijo EM, Landeau B, La Joie R, Mevel K, Mézenge F, Perrotin A (2013). Relationships between years of education and gray matter volume, metabolism and functional connectivity in healthy elders. Neuroimage.

[25] Menon V. Salience Network. Brain Mapp An Encycl Ref. 2015;2:597–611.

[26] Folstein MF, Folstein SE, McHugh PR (1975). “Mini-mental state”. A practical method for grading the cognitive state of patients for the clinician. J Psychiatr Res.

[27] Knopman David S., Petersen Ronald C. (2014). Mild Cognitive Impairment and Mild Dementia: A Clinical Perspective. Mayo Clinic Proceedings.

[28] Kerti L, Witte AV, Winkler A, Grittner U, Rujescu D, Flöel A (2013). Higher glucose levels associated with lower memory and reduced hippocampal microstructure. Neurology.

[29] Köbe T, Witte AV, Schnelle A, Grittner U, Tesky VA, Pantel J (2016). Vitamin B-12 concentration, memory performance, and hippocampal structure in patients with mild cognitive impairment. Am J Clin Nutr.

[30] Lezak MD, Howieson DB, Loring DW, Hannay HJ, Fischer JS. Neuropsychological assessment. 4th ed; New York, Oxford: Oxford University Press; 2004.

[31] Reitan R (1958). Validity of the Trail Making Test as an indicator of organic brain damage. Percept Mot Skills.

[32] Woodard JL, Axelrod BN (1987). Wechsler Memory Scale–Revised. Psychol Assess.

[33] Koss Elisabeth, Ober Beth A., Delis Dean C., Friedland Robert P. (1984). The Stroop Color-Word Test: Indicator of Dementia Severity. International Journal of Neuroscience.

[34] Wechsler D. *WAIS-R manual: Wechsler adult intelligence scale-revised*. Psychological Corporation. 1981.

[35] Harth S, Müller SV, Aschenbrenner S, Tucha O, Lange KW (2004). Regensburger Wortflüssigkeits-Test (RWT). Z Neuropsychol.

[36] Schmidt P, Gaser C, Arsic M, Buck D, Förschler A, Berthele A (2012). An automated tool for detection of FLAIR-hyperintense white-matter lesions in multiple sclerosis. Neuroimage.

[37] Lange Catharina, Suppa Per, Mäurer Anja, Ritter Kerstin, Pietrzyk Uwe, Steinhagen-Thiessen Elisabeth, Fiebach Jochen B., Spies Lothar, Buchert Ralph (2016). Mental speed is associated with the shape irregularity of white matter MRI hyperintensity load. Brain Imaging and Behavior.

[38] Malone Ian B., Leung Kelvin K., Clegg Shona, Barnes Josephine, Whitwell Jennifer L., Ashburner John, Fox Nick C., Ridgway Gerard R. (2015). Accurate automatic estimation of total intracranial volume: A nuisance variable with less nuisance. NeuroImage.

[39] D’Agostino RB, Vasan RS, Pencina MJ, Wolf PA, Cobain M, Massaro JM (2008). General cardiovascular risk profile for use in primary care: The Framingham heart study. Circulation.

[40] Whitfield-Gabrieli S, Nieto-Castanon A (2012). *Conn*: A functional connectivity toolbox for correlated and anticorrelated brain networks. Brain Connect.

[41] Behzadi Yashar, Restom Khaled, Liau Joy, Liu Thomas T. (2007). A component based noise correction method (CompCor) for BOLD and perfusion based fMRI. NeuroImage.

[42] Cole MW, Repovs G, Anticevic A (2014). The frontoparietal control system: a central role in mental health. Neuroscientist.

[43] Bressler Steven L., Menon Vinod (2010). Large-scale brain networks in cognition: emerging methods and principles. Trends in Cognitive Sciences.

[44] Franzmeier N, Caballero MÁA, Taylor ANW, Simon-Vermot L, Buerger K, Ertl-Wagner B (2017). Resting-state global functional connectivity as a biomarker of cognitive reserve in mild cognitive impairment. Brain Imaging Behav.

[45] Muthén L, Muthén B. Mplus user’s guide. 8th ed: Los Angeles CA: Muthén & Muthén 2017.

[46] Hessler J, Jahn T, Kurz A, Bickel H (2013). The MWT-B as an estimator of premorbid intelligence in MCI and dementia. Z Neuropsychol.

[47] Flöel A, Witte a V, Lohmann H, Wersching H, Ringelstein EB, Berger K (2008). Lifestyle and memory in the elderly. Neuroepidemiology.

[48] Frey I, Berg A, Grathwohl D, Keul J (1999). Freiburger Fragebogen zur kSrperlichen Aktivit it- Entwicklung, PriJfung und Anwendung. Soz Praventivmed.

[49] Hu LT, Bentler PM (1999). Cutoff criteria for fit indexes in covariance structure analysis: conventional criteria versus new alternatives. Struct Equ Model.

[50] Cook RD (1977). Detection of influential observation in linear regression. Technometrics.

[51] Klein A, Moosbrugger H (2000). Maximum likelihood estimation of latent interaction effects with the LMS method. Psychometrika.

[52] Smith EE, Salat DH, Jeng J, McCreary CR, Fischl B, Schmahmann JD (2011). Correlations between MRI white matter lesion location and executive function and episodic memory. Neurology.

[53] Gunning-Dixon FM, Raz N (2000). The cognitive correlates of white matter abnormalities in normal aging: a quantitative review. Neuropsychology.

[54] Franzmeier N, Hartmann JC, Taylor ANW, Caballero MÁA, Simon-Vermot L, Buerger K (2017). Left frontal hub connectivity during memory performance supports reserve in aging and mild cognitive impairment. J Alzheimers Dis.

[55] Franzmeier N, Düzel E, Jessen F, Buerger K, Levin J, Duering M, et al. Left frontal hub connectivity delays cognitive impairment in autosomal-dominant and sporadic Alzheimer’s disease. Brain. 2018;141:1186–200. 10.1093/brain/awy008 PMC5888938 29462334

[56] Arenaza-Urquijo EM, Wirth M, Chételat G (2015). Cognitive reserve and lifestyle: moving towards preclinical Alzheimer’s disease. Front Aging Neurosci.

[57] Menon V, Uddin LQ (2010). Saliency, switching, attention and control: a network model of insula function. Brain Struct Funct.

[58] Liu J, Xia M, Dai Z, Wang X, Liao X, Bi Y, et al. Intrinsic brain hub connectivity underlies individual differences in spatial working memory. Cereb Cortex. 2017;27:5496–508. 10.1093/cercor/bhw317 28334075

[59] Pinter Daniela, Enzinger Christian, Fazekas Franz (2015). Cerebral small vessel disease, cognitive reserve and cognitive dysfunction. Journal of Neurology.

[60] Arenaza-Urquijo EM, Vemuri P (2018). Resistance vs resilience to Alzheimer disease. Neurol Int.

[61] Antonenko D, Külzow N, Sousa A, Prehn K, Grittner U, Flöel A (2018). Neuronal and behavioral effects of multi-day brain stimulation and memory training. Neurobiol Aging.

[62] Meinzer Marcus, Lindenberg Robert, Phan Mai Thy, Ulm Lena, Volk Carina, Flöel Agnes (2015). Transcranial direct current stimulation in mild cognitive impairment: Behavioral effects and neural mechanisms. Alzheimer's & Dementia.

[63] Passow S, Thurm F, Li SC. Activating developmental reserve capacity via cognitive training or non-invasive brain stimulation: potentials for promoting fronto-parietal and hippocampal-striatal network functions in old age. Front Aging Neurosci. 2017;9:33. 10.3389/fnagi.2017.00033 PMC5322263 28280465

[64] Bullmore Ed, Sporns Olaf (2009). Complex brain networks: graph theoretical analysis of structural and functional systems. Nature Reviews Neuroscience.

[65] Taylor ANW, Kambeitz-Ilankovic L, Gesierich B, Simon-Vermot L, Franzmeier N, Araque Caballero M, et al. Tract-specific white matter hyperintensities disrupt neural network function in Alzheimer’s disease. Alzheimer’s Dement. 2017;13:225–35. 10.1016/j.jalz.2016.06.2358 PMC5319922 27432800

