# Supplementary material for: Functional connectivity in cognitive control networks mitigates the impact of white matter lesions in the elderly
Source: Alzheimers Res Ther. 2018 Oct 27;10:109. doi: 10.1186/s13195-018-0434-3 (PMC6204269; doi:10.1186/s13195-018-0434-3)
Supplement: Supplementary file 1 — Table S1. Measurement models. Note: given that the salience network and reserve model only contain three nodes/indicators, model fit cannot be provided as the model is just identified. However, factor loadings are considerably high between 0.53 and 0.81. CFI comparative fit index, RMSEA root mean square error of approximation, SRMR standard root mean square residual. Table S2. Movement parameters depicted as mean and standard deviation. Independent sample test for group differences. Table S3. Cognitive scores are depicted as mean and standard deviation (SD) with range for the entire sample and by group. Table S4. Results of seed-to-voxel based regression analysis of CR in each network seed. Coordinates are provided in MNI coordinates (xyz). Significant clusters were extracted at a cluster-level threshold of p < 0.05, FDR-corrected for multiple comparison, and a voxel-level threshold of p < 0.005. LP lateral parietal, ACC anterior cingulate cortex, MPFC medial prefrontal cortex. (DOCX 25 kb) [file 13195_2018_434_MOESM1_ESM.docx]

**Additional file 1**

**Table S1**

CFI – Comparative Fit Index; RMSEA – Root Mean Square Error of Approximation; SRMR – standard root mean square residual; Note: Given that the salience network and reserve model only contain 3 node/indicators, model fit cannot be provided as the model is just identified. However, factor loadings are considerably high between 0.53 and 0.81.

**Table S2**

Movement parameters depicted as mean and standard deviation. Independent sample test for group differences.

**Table S3.** Cognitive Scores are depicted as mean and standard deviation (SD) with range for the entire sample and by group.

**Table S4.** Results of seed- to-voxel based regression analysis of CR in each network seed. Coordinates are provided in MNI coordinates (xyz). Significant clusters were extracted at a cluster-level threshold of *p* < 0.05, FDR corrected for multiple comparison, and a voxel-level threshold of *p* < 0.005. Abbreviations: LP lateral parietal; ACC, anterior cingulate cortex; MPFC, medial prefrontal cortex.

# Supplement

| **Table S1.** Measurement models | | | | | |
| --- | --- | --- | --- | --- | --- |
| Factors | χ^2^ | *df* | CFI | RMSEA | SRMR |
| Cognition | 78 | 29 | .951 | .086 | .057 |
| Fronto Parietal | 3.8 | 3 | .998 | .035 | .024 |
| Default Mode | 22 | 6 | .950 | .108 | .039 |

**Table S2.** Movement parameters

| Parameters | HO  (n=140) | MCI  (n=90) | *p* |
| --- | --- | --- | --- |
| Invalid Scans  M (SD) | 1.7  (3.6) | 1.6  (2.7) | .68 |
| Mean Motion  M (SD) | .05  (.05) | .06  (.07) | .19 |

**Table S3.**

|  | **Total Sample**  **(N=230)** | | | **HO**  **(N=140)** | | | **MCI**  **(N=90)** | | |
| --- | --- | --- | --- | --- | --- | --- | --- | --- | --- |
|  | Mean (SD) | Min | Max | Mean (SD) | Min | Max | Mean (SD) | Min | Max |
| VLM Total Recall | 49 (11) | 20 | 75 | 52 (10) | 30 | 75 | 43 (10) | 20 | 68 |
| VLMT delayed recall | 9 (4) | 0 | 15 | 10 (3) | 1 | 15 | 7 (4) | 0 | 14 |
| VLMT Recognition | 10 (5) | -11 | 15 | 12 (3) | -3 | 15 | 8 (6) | -11 | 15 |
| Trail Making test A | 39.89 (16.00) | 19 | 127 | 35.59 (11.72) | 20 | 87 | 46.57 (19.23) | 19 | 127 |
| Trail Making test B | 97.33 (50.54) | 33 | 300 | 86.00 (41.08) | 33 | 300 | 114.95 (58.50) | 41 | 300 |
| Stroop Interference | 89.17 (27.79) | 51 | 290 | 82.29 (16.85) | 51 | 168 | 99.95 (36.85) | 62 | 290 |
| Digit Span fw | 7 (2) | 2 | 12 | 8 (2) | 4 | 12 | 7 (2) | 2 | 11 |
| DigitSpan bw | 6 (2) | 2 | 12 | 6 (2) | 2 | 12 | 6 (2) | 2 | 11 |
| PhonemicFluency | 14 (5) | 2 | 30 | 16 (5) | 5 | 30 | 10 (4) | 2 | 20 |
| Phonemic Flexibility | 13 (4) | 4 | 23 | 14 (4) | 4 | 23 | 12 (4) | 5 | 23 |

**Table S4.**

| Regions | Number of Voxel | *p-corrected* | *T* | Peak MNI Coordinates | | |
| --- | --- | --- | --- | --- | --- | --- |
|  |  |  |  | x | y | z |
| ***Fronto-parietal Network seed: LP*** | | |  |  |  |  |
| Frontal Pole Left | 485 | 0.00 | 4.40 | -48 | +56 | -06 |
| ***Salience network seed: ACC*** | | |  |  |  |  |
| Frontal Medial Cortex | 801 | .001 | 3.82 | -12 | +38 | -04 |
| ***Default mode network seed: MPFC*** | | |  |  |  |  |
| Lateral Occipital Cortex, L | 249 | .011 | 4.53 | -58 | 66 | +18 |
| Lateral Occipital Cortex, R | 205 | .059 | 3.90 | +54 | -58 | +14 |
